# Pain comorbidities – understanding and treating the complex patient: IASP Press, 2012

**DOI:** 10.1186/1129-2377-14-38

**Published:** 2013-04-29

**Authors:** Simona Sacco

**Affiliations:** 1 Department of Neurology, University of L'Aquila, Piazzale Salvatore Tommasi 1, 67100, L'Aquila, Italy

## Abstract

**Book details:**

Maria Adele Giamberardino, Troels Staehelin Jensen

Pain Comorbidities. Understanding and Treating the Complex Patient.

IASP Press, Seattle; 2012

507 pages, ISBN 978-0-931092-92-3

## 

This book provides extensive information about Pain, with a particular focus on comorbidities, and is referred to a wide audience of physicians because of the ubiquitous diffusion of this complaint in almost all fields of medicine. The presence of more than one medical condition in the same patient in not uncommon, even more when considering pain, and is probably increasing as a consequence of aging of the population. Proper knowledge of the wide spectrum of pain comorbidities can expand our knowledge of symptoms interactions and of their implications for diagnosis and management. Indeed, the choice of the most appropriate treatment should take into account comorbidities as their treatment can not ignore the presence of pain. Clinicians involved in the management of patients who complain of pain should be aware of notions and concepts reported in this book.

The book is structured into the following parts: I - General aspects, epidemiology and models; II - Concurrent pain and non-pain conditions; and III - Management aspects. Part I refers to epidemiology of pain, animal and human models of pain, neurobiological approach to chronic pain and comorbidities, genetic factors, hormonal contributions, role of the immune system and influence of psychosocial environment thus providing the basis to understand pain comorbidities. Part II refers to the major conditions that may be comorbid with pain including hypertension, diabetes, obesity, cardiovascular diseases, metabolic and inflammatory diseases, affective disorders, myofascial pain syndromes, visceral pain syndromes, fibromyalgia, and joint diseases. Lastly, Part III discusses factors involved in treatment of patients such as multidisciplinary pain centers, pharmacological approach, antidepressants, physical training and rehabilitation, and psychological management. All chapters are written by well-known and qualified contributors.

I recommend this book, as an opportunity to get a better knowledge of the topic, not only to clinicians directly involved in pain management but also to general physicians, internists, anesthesiologists, neurologists, psychiatrists, and cardiologists.

## Competing interests

The author declare that she has no competing interests.

